# Combined treatment using repurposed synthetic peptide desmopressin and bevacizumab as a potential antiangiogenic strategy in osteosarcoma

**DOI:** 10.3389/fmed.2026.1864843

**Published:** 2026-07-01

**Authors:** Luisina María Solernó, Candela Llavona, Zahira Yasmine Saud, Mariana Carolina Onassis, María Florencia Gottardo, Martín Manuel Ledesma, Daniel Fernando Alonso, Juan Garona

**Affiliations:** 1 Centro de Oncología Molecular y Traslacional (COMTra), Departamento de Ciencia y Tecnología, Universidad Nacional de Quilmes, Buenos Aires, Argentina; 2 Unidad de Investigación Biomédica en Cáncer (IBioCan), Centro de Medicina Traslacional, Hospital de Alta Complejidad en Red El Cruce “Dr. Néstor Kirchner, ” Buenos Aires, Argentina; 3 Consejo Nacional de Investigaciones Científicas y Técnicas (CONICET), Buenos Aires, Argentina; 4 Hospital Interzonal de Niños “Eva Perón,” Catamarca, Argentina; 5 Hospital de la Madre y el Niño, La Rioja, Argentina; 6 Hospital de Alta Complejidad en Red El Cruce “Dr. Néstor Kirchner, Buenos Aires, Argentina

**Keywords:** adjuvant therapy, angiogenesis, AVPR2, bevacizumab, bone tumor, desmopressin, osteosarcoma

## Abstract

**Background:**

Osteosarcoma (OSA) is the most common primary malignant bone tumor and is characterized by high mortality, early metastatic dissemination, and extensive vascularization. Although overexpression of Vascular Endothelial Growth Factor A (VEGF-A) is associated with poor prognosis, clinical responses to the anti-VEGF-A monoclonal antibody Bevacizumab (BEVA) have been limited. Desmopressin (dDAVP), a hemostatic agent that acts as a selective arginine vasopressin receptor 2 (AVPR2) agonist, has demonstrated antitumor and angiostatic properties in other malignancies, however, its role in OSA remains incompletely characterized. This study evaluated the therapeutic potential of dDAVP as a coadjuvant strategy to enhance BEVA efficacy in OSA.

**Methods:**

Bioinformatic analyses of AVPR2 expression and its associations with tumor aggressiveness, immune and stromal infiltration markers, and clinical outcomes were performed using TCGA-SARC and TARGET-OS datasets. Endothelial proliferation, migration, and morphogenesis were assessed in HmVEC-L and HMEC-1 microvascular cells. Antitumor activity was evaluated in human (MG-63) and murine (K7M3) OSA models. *In vivo* activity was assessed using xenogeneic and syngeneic models of tumor growth, metastasis, and tumor-associated angiogenesis. Combined dDAVP + BEVA therapy was evaluated *in vitro* and *in vivo*. Histopathological endpoints included mitotic index, desmoplasia, vimentin expression, and tumor necrosis.

**Results:**

Exploratory transcriptomic analyses revealed that AVPR2 expression was inversely associated with proangiogenic, prosurvival, and prometastatic gene signatures, while positively correlating with immune and stromal infiltration markers. Elevated AVPR2 expression was also associated with improved survival in both the pan-sarcoma cohort and a pediatric OSA subset. dDAVP significantly reduced pulmonary metastasis and OSA-driven vascularization *in vivo* and inhibited endothelial proliferation, migration, and morphogenesis *in vitro*. Under tumor-conditioned conditions, dDAVP enhanced the antivascular activity of BEVA. Combined treatment with clinically relevant doses of dDAVP and BEVA significantly inhibited OSA xenograft progression without overt toxicity and favorably modulated histopathological markers of tumor aggressiveness.

**Conclusion:**

These findings support further translational evaluation of dDAVP as a potential adjuvant therapy in OSA, particularly in combination with BEVA. By targeting complementary angiogenesis-related mechanisms, this dual antiangiogenic strategy may improve therapeutic efficacy in OSA.

## 1 Introduction

Osteosarcoma (OSA) is the most prevalent primary bone malignancy, predominantly affecting children and young adults, and ranks as the eighth most common pediatric cancer, accounting for approximately 2.4% of all tumors diagnosed in this population (1, 2). Management of this highly aggressive sarcoma relies on surgical resection of primary and metastatic lesions combined with multi-agent chemotherapy. However, clinical outcomes remain suboptimal (3). OSA is characterized by rapid tumor growth, early metastatic dissemination, predominantly to the lungs, and a highly vascularized microenvironment (4). Numerous studies have demonstrated that tumor expression of Vascular Endothelial Growth Factor A (VEGF-A), a driver molecule of the angiogenic process, correlates with metastatic disease progression and poor response to chemotherapy in OSA (5, 6). Driven by the stagnation in survival outcomes and by advances in the understanding of molecular mechanisms underlying OSA progression, substantial efforts over the past decade have focused on developing novel targeted therapies to complement multimodal treatment strategies, many of which specifically aim to disrupt tumor angiogenesis (7). Among these is bevacizumab (BEVA), a humanized recombinant monoclonal antibody of the IgG1 type that binds to and inhibits VEGF-A by preventing its interaction with its receptor (VEGFR) on the surface of endothelial cells. In OSA preclinical studies BEVA showed highly promising results. However, across multiple clinical trials evaluating BEVA as an adjuvant therapy in OSA, only partial responses were observed in histological and vascular parameters, while primary survival endpoints were not achieved, either as monotherapy or in combination with standard chemotherapy (8–10). Moreover, it exhibited frequent wound healing complications along with increased risk of postoperative bleeding, an adverse outcome considering the inherent morbidity associated with radical surgical resection in OSA tumors (11, 12). In this context, clinical experience with BEVA in OSA highlights the limitations of single-target antiangiogenic strategies, suggesting that multi-step inhibition through the combination of complementary angiostatic and cytostatic agents may represent a more effective therapeutic approach.

Consequently, drug repurposing in oncology, the oncological use of drugs originally developed for other pathologies, emerges as a promising alternative to implement in a disease with few novel therapeutic options. Since safety profiles are already established, the testing phases are facilitated, allowing for accelerated clinical implementation (13, 14). In recent years, our research group has proposed and explored the use of the hemostatic drug desmopressin (1-deamino-8-D-arginine vasopressin, dDAVP), originally used in patients with bleeding disorders (15), as a novel therapeutic targeted agent in various aggressive neoplasms, including neuroendocrine tumors, hormone-independent breast cancer and colorectal cancer (16–19). In contrast to the natural hormone vasopressin, which interacts with all vasopressin receptor subtypes, the synthetic analogue dDAVP acts as a selective agonist of the arginine vasopressin receptor 2 (AVPR2), which is expressed in both microvascular endothelium and tumor tissue, triggering different antitumor mechanisms (17–19). In an exploratory study, our group first demonstrated the direct tumor cell–mediated cytostatic activity of dDAVP, showing induction of apoptosis, mitotic arrest, and reduced migratory capacity of AVPR2-expressing OSA cells. In addition, AVPR2 immunoreactivity was detected in approximately 70% of chemotherapy-naïve human OSA biopsies, supporting the clinical relevance of this target (20). However, despite these findings, the contribution of dDAVP to OSA-associated angiogenesis remains poorly understood, and its integration with targeted therapies has not been systematically investigated, representing a significant gap in the development of effective translational strategies for this disease.

In this study, we first evaluated the prognostic relevance of AVPR2 and its association with protumoral markers were assessed using publicly available transcriptomic datasets from sarcoma clinical samples, including OSA, with particular emphasis on stroma-related processes such as angiogenesis, metastatic dissemination, and immune infiltration. Second, this study aimed to characterize the *in vitro* and *in vivo* inhibitory effects of dDAVP on OSA-associated angiogenesis and metastasis. Finally, based on these findings, we explored a coadjuvant therapeutic strategy combining dDAVP with the antiangiogenic agent BEVA.

## 2 Materials and methods

### 2.1 Drugs

1-deamino-8-D-arginine vasopressin was obtained from Kedrion Laboratories (EMOSINT 20 μg/ml) and monoclonal IgG1 antibody BEVA was kindly provided by Elea-Phoenix Laboratories (Bevax^®^- 25 mg/ml). To achieve work concentrations, both were diluted in phosphate buffered saline (PBS) as vehicle. All control groups received PBS as vehicle.

### 2.2 Cell lines and culture conditions

MG-63 is a human cell line (RRID:CVCL_0426) with fibroblast morphology isolated from the bone of a white, 14-year-old male patient with OSA. It was grown in Dulbecco’s modified Eagle’s medium (DMEM) plus 10% fetal bovine serum (FBS), 2 mM glutamine and 80 μg/ml gentamicin in monolayer culture, at 37 °C in a humidified atmosphere of 5% CO_2_. Murine OSA K7M3 cell line (RRID:CVCL_XG68) was grown in DMEM: F12 medium with the same supplements listed before. It is an aggressive and highly metastatic cell line described by Gordon et al. (21) that was originally developed by cycling the K7M2 parental cell line in mice via tail vein injection and harvesting the resulting lung metastases for further culture. HMEC-1 (CLS Cat# 304064, RRID:CVCL_0307) is an immortalized human dermal microvascular endothelial cell line isolated from the endothelium of the foreskin of a male patient. Its culture conditions were the same as stated for MG-63 cells. HmVEC-L (Lonza CC-2527) is a human lung microvascular endothelial cellular model, and it was grown in EBM-2 supplemented with EGM-2 MV BulletKit at 37 °C in a humidified atmosphere of 5% CO_2_. HmVEC-L cells were used in passages 8–12.

### 2.3 Bioinformatic studies

GEPIA2^1^ (Gene Expression Profiling Interactive Analysis 2 (RRID:SCR_026154)) and TIMER2.0^2^ (Tumor IMmune Estimation Resource (RRID:SCR_018737), transcriptomic analysis tools that contain RNA-sequencing information from The Cancer Genome Atlas (RRID:SCR_003193 - TCGA)), were used. Specifically, sarcoma (SARC) database, containing 257 patient cases diagnosed with different sarcomas, including OSA, was analyzed. Correlation between AVPR2 and different specific genes related to cell cycle progression, apoptosis, metastases and angiogenesis was assessed by individual genes analysis using TIMER2.0. Also, correlation between AVPR2 and immune infiltration was studied. To account for the confounding effects of sample heterogeneity, partial Spearman’s correlation was utilized to assess the association between AVPR2 expression and stromal cell abundance, applying purity adjustment to ensure the robustness of the identified correlations. Nominal *p*-values were adjusted for multiple comparisons using the Benjamini-Hochberg false discovery rate method. Associations were considered statistically significant after correction when the adjusted *p*-value/FDR was < 0.05. To deepen this study, a pooled gene analysis using GEPIA2 was performed to study the correlation between AVPR2 and genes related to the biological processes mentioned before.

Also, AVPR2 prognostic impact in overall survival (OS) and disease-free survival (DFS) was studied using GEPIA2 and the SARC-TCGA database. Spearman’s rank correlation coefficient was used for this analysis. The RNA-seq database TARGET-OS was downloaded using the TCGAbiolinks R package (R 4.4.2) and processed with its recommended pipeline, including STAR alignment, count estimation, preprocessing, normalization, and gene filtering. Clinical metadata (demographics, diagnosis, treatment, survival) were retrieved and merged by patient barcode. For downstream analyses, we restricted the cohort to patients with vital_status = “Dead” (*n* = 29) and used normalized AVPR2 (ENSG00000126895) expression, which was log_2_-transformed [log_2_(count + 1)]. AVPR2 expression was dichotomized within the deceased cohort at the cohort mean of log_2_(ExprAVPR2 + 1) to define “High” versus “Low.” Survival time was calculated as days_to_death/30.4 (months). OS was defined as the time from diagnosis to death. Survival analyses were performed using the Kaplan–Meier method, and group differences were tested with the log-rank test. Median survival times and 95% confidence intervals (95% CI) were computed. All analyses were conducted in R (version 4.4.2) using the survival and survminer packages. To explore clinical stratification, we created age groups (<15 vs. >15 years) and a two-level mortality-time risk variable: <20 months (high risk) and >20 months (low risk). Group differences in AVPR2 expression across mortality-time strata were strictly evaluated in pediatric population (patients < 15 years; *n* = 19) using χ^2^ tests with Monte-Carlo simulation of *p*-values. Pearson standardized residuals were then plotted to identify cells driving the lack of homogeneity fit; values above 2 or below −2 imply significantly higher or lower frequencies than expected.

### 2.4 Tumor and microvascular cell growth

To evaluate dDAVP direct effect on OSA or microvascular cell proliferation 4 × 10^3^ K7M3 or 4 × 10^3^ HMEC-1 cells were plated in 96 well flat bottom plates in complete medium, allowed to attach, and 24 h later treated with dDAVP (0.1, 1, or 10 μM) or PBS for 72 h. At the end of the experiment, cultures were fixed with methanol, stained with 0.5% crystal violet and absorbance was measured at 595 nm. The optical density of PBS- treated control cells was taken as 100% of cell growth.

### 2.5 Endothelial cell chemotaxis

Endothelial migration was assessed using the Transwell^®^ migration assay and the HMEC-1 cell line (22). After a 24-h starvation, 5 × 10^4^ HMEC-1 cells were seeded into 8 μm pore inserts in 24- well plates in serum- free growth medium. The lower chamber was filled with DMEM containing 10% FBS, which was used as a chemoattractant. After 24- h incubation with dDAVP (0.1 or 1 μM) cells in the upper surface of the membranes were gently removed with cotton swabs. Cells that migrated to the lower surface were fixed with methanol and stained with 0.5% crystal violet. Migrated cells in five randomly selected ×400 high-power fields (HPF) were counted and normalized to control.

### 2.6 Endothelial cell morphogenesis assay

In order to conduct an *in vitro* vascular cell morphogenesis assay 24 well flat bottom plates were covered with Matrigel™ (growth factor reduced, high concentration, Corning) and incubated at 37°C for 30 min. HmVEC-L cells were utilized given their capacity to form tubular like structures (18). 1 × 10^5^ cells were plated per well in complete medium with dDAVP (0.1 μM) or PBS for control group and allowed to grow overnight (16 h). Tube quantification was carried out counting the number of vessels sprouts in 10 × 100 HPF (23, 24).

### 2.7 TCM-stimulated microvascular endothelial cell growth

To obtain the tumor conditioned media (TCM) of human OSA MG-63 cells were plated in complete medium and, after 24 h, the medium was replaced with a serum-free DMEM. After 24 h, the culture supernatant was collected and centrifuged at 5,000 rpm for 10 min to remove detached cells and cellular debris. Simultaneously, HMEC-1 cells were seeded into 96 well plates (4 × 10^3^ cells/well) in complete medium. After 24 h, the depleted culture medium was removed and replaced with 150 μL fresh medium supplemented with 2% FBS (final concentration), plus 50 μL of MG-63 TCM in addition to BEVA (25 μg/ml), dDAVP (1 μM) or BEVA plus dDAVP (1 μM). After a 48 h incubation, cell growth was determined using the crystal violet method described before.

### 2.8 Animals

Outbred athymic female N:NIH(S)-nu (RRID:IMSR_CRL:490) mice and inbred immunocompetent male BALB/c AnN (RRID:MGI:2161072) aged 8 weeks with a weight of approximately 20 g, were obtained from *Universidad Nacional de La Plata* Animal Facility. Upon arrival at the Animal Facility of Universidad Nacional de Quilmes, mice were randomly assigned to the different experimental groups and housed at a density of 4–6 animals per cage. Appropriate environmental enrichment was provided according to our Animal Care Committee. For each *in vivo* protocol, animal body weights were assessed following randomization to verify balanced group allocation and to exclude the presence of significant baseline weight differences among cages and experimental groups before treatment initiation. Food and water were provided *ad libitum*, general health status and wellbeing of the animals was monitored daily and a rewards of sunflower seeds was offered after each administration. Animal protocols were approved by our institutional Animal Care Committee (Resolution CD CyT No. 075/14, Protocol codes: 011–15/010-15/007-15). Animal studies were reported in accordance with ARRIVE guidelines. Sample sizes for the experimental metastasis, modified Matrigel plug, and xenograft progression studies were established based on previous preclinical studies published by our group and others employing comparable animal models, experimental designs, and outcome measures (20, 25, 26). Group sizes were subsequently validated using the GRANMO sample size calculator^3^. For this purpose, after establishing the number of experimental groups, the parameters were set at an c\ error of 0.05 (95% confidence level) and a β error of 0.2 (80% power), accounting for an anticipated animal attrition rate of 10%, with standard deviations (SD) tailored to each specific experimental protocol. Control groups received corresponding vehicles.

### 2.9 OSA xenograft progression

To generate human OSA xenografts 5 × 10^6^ MG- 63 cells, in a 150 μl- suspension in 1:0.5 volume ratio mixture of DMEM and Matrigel™ (Corning), were injected subcutaneously (s.c.) in athymic mice. Tumors were measured periodically with a caliper and tumor volume was calculated (0.52 × width^2^ × length). Animal weights and tumor growth rates (TGR, representing the slopes of the linear regressions of the tumor volumes over time) were also assessed. Treatment schedules started one week after tumor cell injection, as primary tumors were detected by palpation. When the first tumors from vehicle-treated animals reached humane tumor burden limits (>300 mm^3^), and before ulceration and skin infiltration were observed, animals were euthanized by cervical dislocation and tumors were removed for further histopathological analysis. The clinically relevant dosing scheme used for dDAVP was 12 μg/kg i.v., three times per week, and was obtained after “human to mouse” dose conversion following the Food and Drug Administration (FDA) guidelines (reference human dose of 1 μg/kg i.v. multiplied by 12.3) (20, 26, 27). *In vivo* antitumoral activity of dDAVP in addition to BEVA was assessed for 4 weeks. BEVA was administered at 5 mg/kg i.p. dose, two times per week, following a low-toxicity and sustained metronomic scheme (28).

### 2.10 Histopathological analysis

After being resected, tumors were fixed with formalin and processed for further histopathological analysis. All histological analyses were performed in a blinded manner regarding treatment groups. The sampling strategy applied to each histological marker was designed to adequately represent the entire tumor and provide a reliable estimate of the corresponding histopathological endpoint.

Mitotic bodies in viable sections of hematoxylin and eosin (H&E) stained slides were counted in 3–4 randomly-selected HPF (×400) per tumor section to determine mitotic index. Only viable and highly cellular tumor regions were considered for mitotic body quantification.

For tumor necrosis assessment, color brightfield images of two semi-serial H&E-stained tumor sections from each tumor specimen were acquired at ×2.5 magnification using a BioTek Cytation 5 Cell Imaging Multi-Mode Reader (RRID:SCR_019732). This approach was adopted considering the extensive degree of necrosis displayed by the MG-63 xenograft model across all experimental groups and its heterogeneous distribution throughout the tumor tissue. Images were collected using a 4 ×5 grid and the stitching was performed with the “Image Montage” function setting a tile overlapping of 10%. Necrotic area in tumor tissue sections was measured using ImageJ software (RRID:SCR_003070). Tumor necrosis was identified as tissue areas with a marked increase of eosinophilia and quantification of both necrotic area (NA) and viable area (VA) was performed using the “Color Threshold” tool. Tumor necrotic rate (TNR), mentioned in the Results section as “Necrotic fraction,” was then calculated as: “TNR = (NA ×100)/(VA + NA).” In clinical settings, adjustment of the necrotic fraction by integrating both the extent of tumor necrosis and treatment-induced changes in tumor volume has been shown to more accurately predict patient survival and constitutes an independent prognostic factor in OSA (26, 29). Calculation of adjusted tumor necrotic rate (ATNR), mentioned in the Results section as “Relative necrotic index,” was performed using the following equation: “ATNR = 100–(100–TNR) ×RTG,” where RTG stands for group-specific relative tumor growth. RTG was obtained after transforming TG (tumor growth) values, taking the TG of the control group at day 31 (161.3 mm^3^) as “1.” As a result, RTG of 0.54, 0.43, and 0.12 were respectively used for necrosis adjustments in dDAVP, BEVA and dDAVP + BEVA-treated tumor slides.

For desmoplasia analysis, collagen fiber deposition was assessed in 3 or 4 randomly-selected HPF (×400 magnification) per tumor section from viable tissue regions of tumor slides, including peripheral and central areas as well as regions of low and high cellularity, stained with Masson’s trichrome using an optic microscope (Leica). Lesions were graded as null (0), low (1), moderate (2), high (3), or very high (4) depending on the amount of collagen fibers present in the HPF (30).

For anti- human vimentin immunohistochemistry (IHC) sections on silane coated slides were microwaved for antigen retrieval in 0.01 M sodium citrate buffer (pH 6.0) during 30 min, and later incubated with 3% hydrogen peroxide for 10 min to block endogenous peroxidase. Immunostaining was performed using an anti- human vimentin mouse monoclonal antibody (Leica Biosystems Cat# NCL-L-VIM-V9, RRID:AB_564055) at room temperature for 1 h. For visualizing immunoreactivity, a 15- min incubation using the VECTASTAIN^®^ Elite^®^ ABC-HRP Kit (PK-6200, Vector Laboratories Inc.) was conducted. Finally, sections were counterstained with hematoxylin. DAB signal served as an indicator of protein expression and was quantified in 10 to 12 HPF (×1,000 magnification) per group by measuring pixel area using the tool Pixel Classification from QuPath (RRID:SCR_018257).

### 2.11 Modified matrigel plug assay

In order to assess *in vivo* tumor angiogenesis, a mixture containing 400 μl of Matrigel™ (growth factor reduced, high concentration, Corning), heparin (50 U/ml) and 4 × 10^6^ MG-63 cells in 100 μl serum-free DMEM medium was injected subcutaneously into athymic mice. Treatment consisted of one dose of dDAVP 12 μg/kg i.v. 1 h prior to plug establishment and three additional doses every 48 h for 1 week. Animals were sacrificed 7 days after cell injection, plugs were recovered and the degree of vascularization was assessed by the amount of hemoglobin detected in the implants using the Drabkin method. The mean optical density of plugs from the control group was taken as 100% (relative hemoglobin content).

### 2.12 Pulmonary experimental metastases

An experimental model of hematogenous metastatic colonization to the lungs was employed by inoculating 5 × 10^5^ K7M3 cells in FBS-free DMEM:F12 medium into the lateral tail vein of immunocompetent male BALB/c mice. 1 h prior to cell inoculation, animals were treated with dDAVP 12 μg/kg/day i.v. After 24 h, dDAVP was administered again and from this moment on, it was administered three times per week for a total of 6 weeks. When early signs of breathing distress appeared, animals were euthanized by cervical dislocation and lungs were excised, weighted, fixed in Bouin’s solution and quantification of metastatic lesions was performed under a dissecting microscope using a millimeter ruler. Superficial metastatic lesions across all pulmonary lobes were quantified and classified according to size as either <2 or >2 mm in diameter. Then, greater pulmonary lobes were fixed and embedded in paraffin, cut into 4 μm semi-serial sections and mounted on silanized slides. The histological sections were stained using the H&E technique, to allow visualization and quantitative analysis of the degree of metastatic colonization in the different experimental groups. Quantification of intrapulmonary metastatic nodules was conducted using an optic microscope (Leica) and scanning the complete histological section at ×400 magnification. This analysis yielded one histological assessment per biological replicate.

### 2.13 Statistical analysis

Statistical analyses were performed using GraphPad Prism v8.0.2 (RRID:SCR_002798). For comparisons among three or more experimental groups, ANOVA or its non-parametric equivalent, the Kruskal-Wallis test, were utilized. Subsequently, tests such as Dunnett’s test, Tukey’s test, comparison of 95% confidence intervals (CI) for the mean (ANOVA), or Dunn’s test (Kruskal-Wallis) were employed. For comparisons between two groups, either the Student *t*-test or the Mann-Whitney test were used, depending on the parametric or non-parametric distribution of values, respectively. Outlier identification was performed using the “Identify Outliers” tool in GraphPad Prism v8.0.2 (RRID:SCR_002798), applying the recommended ROUT method with a conservative false discovery rate (Q = 1%). Differences were considered statistically significant at a level of *p* < 0.05. Data corresponds to the sum of at least 2 or 3 independent experiments unless stated otherwise.

## 3 Results

**FIGURE 1:**
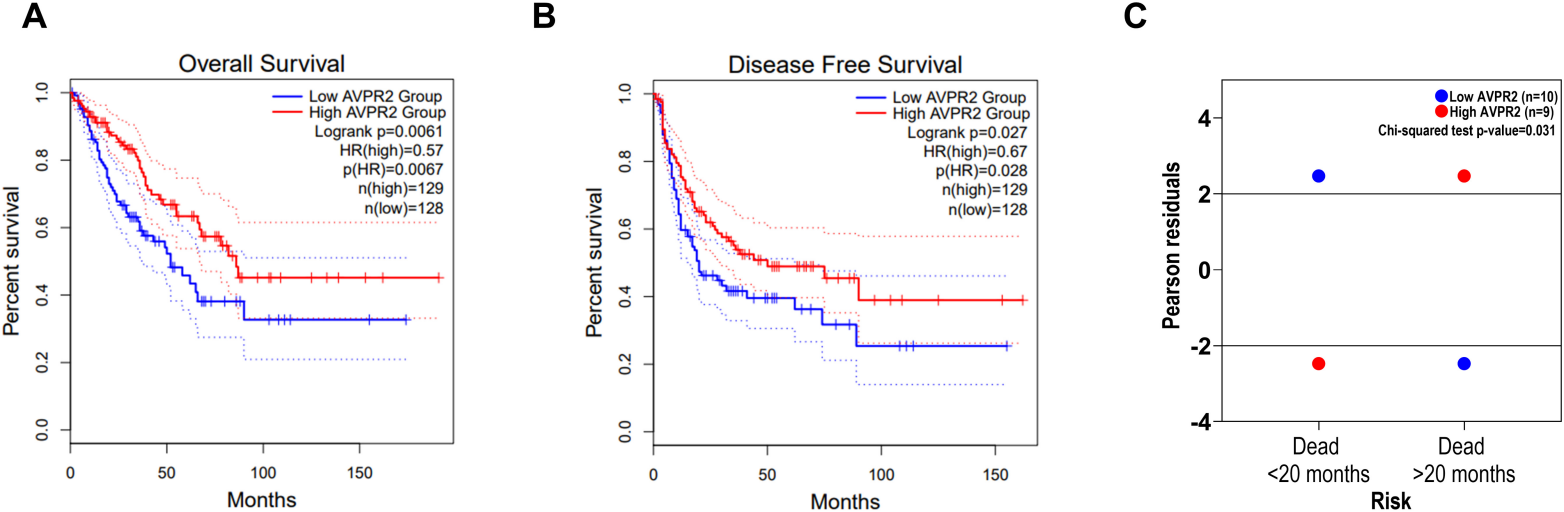
AVPR2 tumor expression prognostic impact on sarcoma and osteosarcoma patients. **(A)** Overall survival of sarcoma patients with low (blue line) or high (red line) tumor expression of AVPR2 receptor. RNA-sequencing data from The Cancer Genome Atlas (TCGA) sarcoma (SARC) dataset (*N* = 257) were analyzed using the GEPIA2 bioinformatic tool. Spearman’s rank correlation coefficient was used for this analysis. **(B)** Disease free survival of sarcoma patients with low (blue line) or high (red line) expression of AVPR2 receptor. RNA-sequencing data from The Cancer Genome Atlas (TCGA) sarcoma (SARC) dataset (*N* = 257) were analyzed using the GEPIA2 bioinformatic tool. Spearman’s rank correlation coefficient was used for this analysis. **(C)** Risk of mortality of osteosarcoma pediatric patients (<15 years) from the TARGET-OS database (*N* = 19) with low (blue dot) or high (red dot) expression of AVPR2 receptor. χ^2^ tests with Monte-Carlo simulation of *p*-values. Differences with a *p*-value < 0.05 were considered statistically significant.

First, the bioinformatic analyses conducted using TIMER2.0 and GEPIA2 relied on the TCGA-SARC pan-sarcoma dataset, which includes 257 cases of aggressive soft-tissue and bone sarcomas, among them OSA. These pathological entities share multiple clinical and biological aspects, such as low survival rates, early metastatic dissemination with lung and bone tropism, a strong dependence on the angiogenic process, and common altered molecular pathways (P53, PI3K/AKT/mTOR, RAS/MAPK), among others (31). Through the assessment of individual gene analysis or pooled gene analysis, a significant negative correlation (Rho < 0; *p* < 0.05) was observed between AVPR2 receptor and genes related to survival, cell cycle progression, apoptosis evasion, metastatic progression, and induction of tumor angiogenesis, in contrast to a positive correlation with apoptosis-inducing genes (Table 1 and Supplementary Table 1). For correlation analyses involving individual genes, the findings and their interpretation remained unchanged after adjustment for tumor purity and FDR. In addition, associations between AVPR2 expression and immune or stromal cell-related gene markers were evaluated. AVPR2 expression was positively correlated with a broad range of immune cell markers, including those associated with natural killer (NK) cells, M1 and M2 macrophages, tumor-associated macrophages (TAMs), total T cells, CD8^+^ T cells, CD4^+^ T cells, Th1, Th2, Th17, regulatory T cells (Tregs), and mast cells, as well as stromal cell populations such as cancer-associated fibroblasts (CAFs), common myeloid progenitors (CMPs), and hematopoietic stem cells (HSCs) (Supplementary Tables 2, 3). The biological roles of these cellular populations in OSA are diverse, complex, and highly context-dependent. Therefore, further validation using OSA-specific transcriptomic datasets will be necessary to confirm these observations in a disease-specific setting. Within the TCGA-SARC pan-sarcoma dataset, elevated AVPR2 expression was associated with a favorable clinical outcome and appeared to correlate with improved overall survival and disease-free survival (Figures 1A, B). Furthermore, analysis of an OSA-specific subset of pediatric patients from the TARGET-OS dataset (<15 years of age and with complete follow-up records) revealed a significant association between higher AVPR2 expression and a lower risk of mortality (Figure 1C). However, given the limitations of the available clinical annotations and follow-up information within the TARGET-OS cohort, this finding should be considered an exploratory, disease-specific transcriptomic observation rather than definitive validation of AVPR2 as a prognostic biomarker in pediatric OSA.

**TABLE 1:** Correlation analysis between arginine vasopressin receptor 2 (AVPR2) expression in sarcoma tumors and gene markers of different biological processes relevant in disease progression using TIMER 2.0.

Biological process involved	Gene markers	None			Purity adjustment		
		Rho	*P*-value	Adjusted *p*-value (BH-FDR)	Rho	*P*-value	Adjusted *p*-value (BH-FDR)
Cell cycle progression	CDK1	−0.334	3.42e-08****	3.25e-07****	−0.297	2.24e-06****	2.13e-05****
	GNL3	−0.276	6.04e-06****	1.91e-05****	−0.283	7.36e-06****	4.33e-05****
	RRS1	−0.202	1.06e-03**	2.01e-03**	−0.235	2.1e-04***	5.70e-04***
	PARP1	−0.222	3.19e-04***	7.58e-04***	−0.228	3.25e-04***	7.72e-04***
Apoptosis induction	STAT5A	0.304	5.85e-07****	2.78e-06****	0.258	4.53e-05****	1.65e-04***
	OLFM4	0.164	8.07e-03**	1.02e-02*	0.131	4.03e-02*	4.08e-02*
	PINK1	0.199	1.29e-03**	2.23e-03**	0.179	5.03e-03**	8.69e-03**
Apoptosis evasion	BIRC5	−0.278	5.48e-06****	1.91e-05****	−0.256	5.22e-05****	1.65e-04***
	API5	−0.191	1.93e-03**	2.82e-03**	−0.166	9.53e-03**	1.29e-02*
	BCL10	−0.102	1.01e-01	1.01e-01	−0.131	4.08e-02*	4.08e-02*
	PRC1	−0.251	4.36e-05****	1.18e-04***	−0.197	2.03e-03**	3.86e-03**
	TRAP1	−0.156	1.16e-02*	1.38e-02*	−0.145	2.32e-02*	2.76e-02*
Metastases	HDAC8	−0.206	8.43e-04***	1.78e-03 **	−0.168	8.58e-03 **	1.26e-02 *
	AMACR	−0.194	1.72e-03**	2.72e-03**	−0.168	8.65e-03**	1.26e-02*
	DLX2	−0.176	4.39e-03**	5.96e-03**	−0.197	1.99e-03**	3.86e-03**
	MKI67	−0.306	4.67e-07****	2.78e-06****	−0.280	9.12e-06****	4.33e-05****
Angiogenesis	VEGFA	−0.356	3.66e-09****	6.95e-08****	−0.322	2.64e-07****	5.02e-03**
	MTOR	−0.142	2.16e-02*	2.41e-02*	−0.148	2.06e-02*	2.61e-02*
	MEK	−0.136	2.84e-02*	3.00e-02*	−0.135	3.47e-02*	3.88e-02*

RNA-sequencing information from The Cancer Genome Atlas (TCGA), sarcoma (SARC) database (*N* = 257). None, correlation without adjustment. Purity, correlation adjusted by purity. Rho > 0 positive correlation, Rho < 0 negative correlation. Spearman’s rank correlation coefficient. Benjamini–Hochberg false discovery rate (BH-FDR) adjusted *p*-values for both the none-corrected and purity-corrected correlation analyses.

*p < 0.05, **p < 0.01, ***p < 0.001, ****p < 0.0001.

Considering the potential therapeutic relevance of AVPR2, the *in vivo* antiangiogenic and antimetastatic activities of dDAVP were investigated as a single-agent intervention in both xenogeneic MG-63 and syngeneic K7M3 OSA models.

**FIGURE 2:**
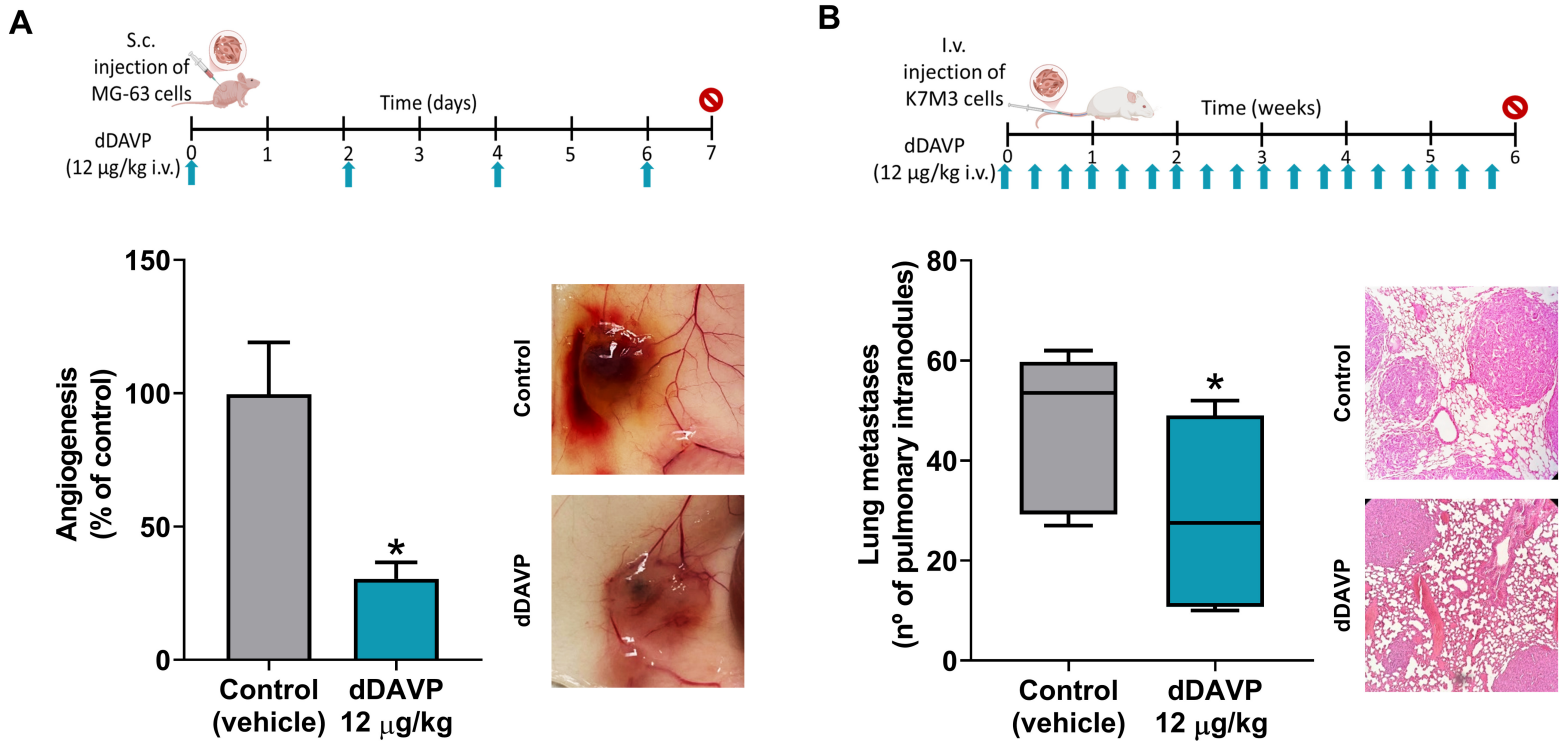
Effects of clinically relevant doses of desmopressin on *in vivo* tumor-driven vascularization and metastatic dissemination. **(A)** Experimental design used to evaluate early angiogenesis induced by OSA MG-63 cells *in vivo* (top). Early vascularization was assessed by quantification of hemoglobin content in the recovered Matrigel™ plugs using the Drabkin’s method. *N* = 4 or 6 animals per experimental group, one independent experiment. Unpaired *t*-test (left). Representative images of subcutaneous vascularized implants (right). **(B)** Experimental design used to evaluate *in vivo* effect of desmopressin (dDAVP, 12 μg/kg i.v.) on pulmonary metastases of K7M3 OSA cells in immunocompetent mice (top). Number of intranodules seen in high power field H&E stained slides. *N* = 6 animals per experimental group, one independent experiment. Unpaired *t*-test (left). Box and whiskers (Min to Max). Representative pictures (right, ×100 magnification). Data are presented as mean ± SEM. *p < 0.05.

Our group previously reported that dDAVP exerts strong and selective cytostatic activity on AVPR2-expressing OSA MG-63 cells and lacks significant antitumor effects on AVPR2-negative cells such as the U2-OS model (even at supra-physiological concentrations of 10 μM) (20). Moreover, K7M3 cells, despite differences in phenotype (mesenchymal versus osteoblastic) and metastatic capacity, exhibit high sensitivity and a comparable dose-dependent response following treatment with dDAVP at concentrations of 0.1, 1, and 10 μM (Supplementary Figure 1). It is widely recognized that OSA progression and metastasis are strongly associated with tumor-driven angiogenesis (32). Using a modified extracellular matrix gel plug assay in nude mice we evaluated the effect of dDAVP on human OSA-related neovascularization *in vivo*. It was observed that short term dDAVP administration at a dose of 12 μg/kg i.v. was capable of inhibiting the vascular response induced by MG-63 human OSA cells, after reducing the hemoglobin content in incipient tumor implants by more than 70% in comparison to the control group (Figure 2A). These findings provide preliminary evidence of altered vascularization by dDAVP in the early stages of OSA establishment.

Considering the clinical relevance of pulmonary metastatic progression in OSA (29), the antimetastatic effect of dDAVP was assessed on intravenously-injected K7M3 cells in immunocompetent BALB/c mice (32). dDAVP was given immediately before and 24 h after tumor cell injection, and following a sustained scheme three times per week, at a dose of 12 μg/kg i.v. After six complete weeks of treatment, dDAVP was capable of significantly reducing OSA metastatic outgrowth, by reducing 37% the number of metastatic intranodules in the lungs, as assessed by histological analysis (Figure 2B). Quantification of superficial pulmonary metastatic lesions according to nodule diameter, together with lung weight measurements, was incorporated as an additional endpoint to provide a more comprehensive assessment of metastatic burden. Notably, dDAVP administration exhibited antimetastatic activity when total metastatic nodules were quantified and, even more prominently, when the analysis was restricted to macrometastatic disease (metastatic lesions > 2 mm in diameter) (Supplementary Figures 2A–C). This antimetastatic effect was further supported by measurements of total lung weight and estimated metastatic burden, calculated as total lung mass minus the average weight of healthy reference lungs (Supplementary Figures 2D, E). No signs of overt toxicity were observed after body weight assessment and general clinical observation (Supplementary Figure 3A).

**FIGURE 3:**
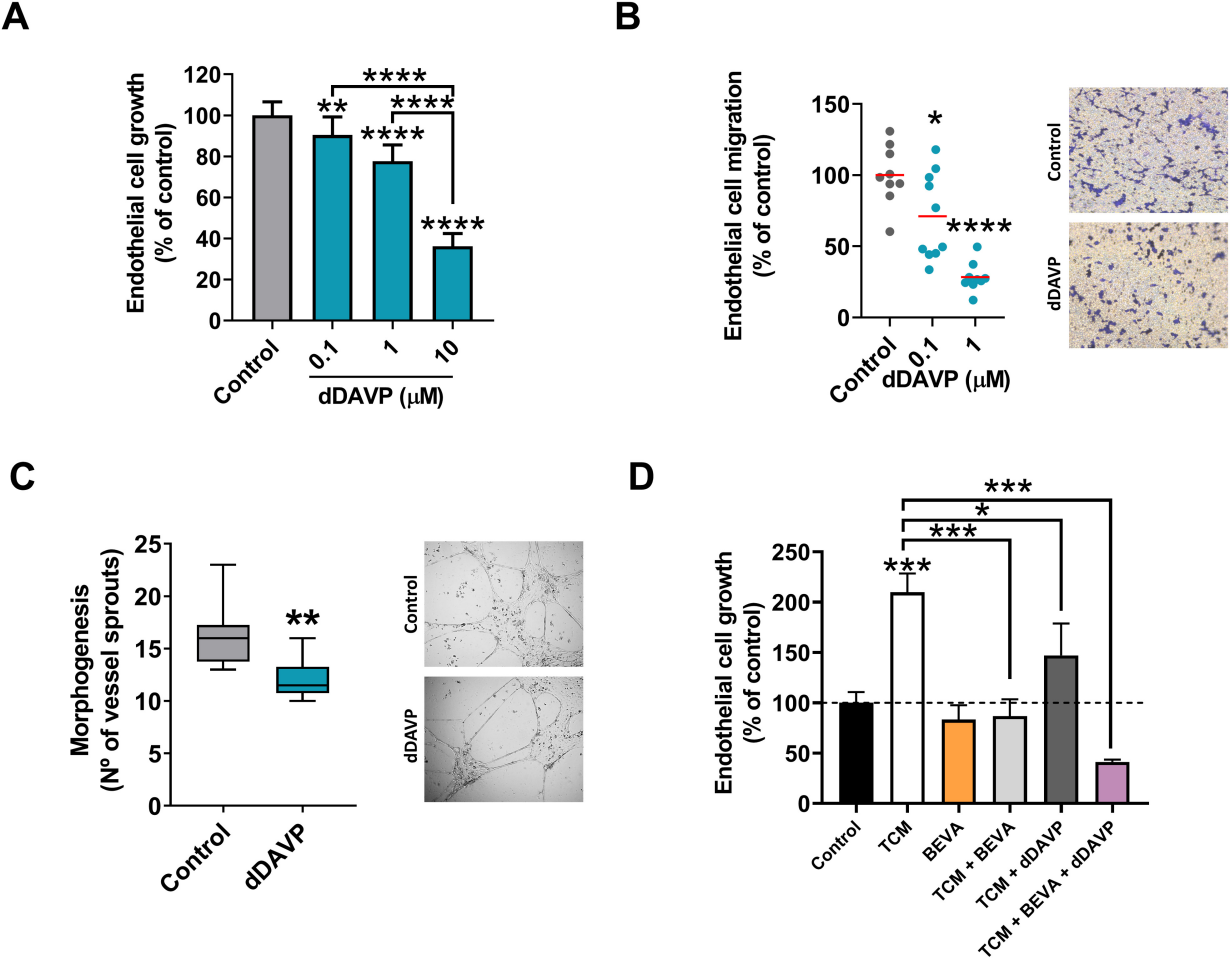
Direct modulation of key cellular processes associated with angiogenesis by desmopressin on microvascular endothelial cells. **(A)** Inhibition of HMEC-1 cell growth by desmopressin (dDAVP, 0.1–10 μM) on exponentially-growing microvascular cultures. ANOVA followed by Tukey’s test. Data are presented as mean ± SD. *N* = 16 technical replicates per experimental group, two independent experiments. **(B)** Impairment of endothelial cell chemotaxis after a 16 h treatment on HMEC-1 cells using desmopressin (dDAVP, 0.1 and 1 μM). Representative pictures of the bottom of the Transwell™ inserts (bottom, ×100 magnification). Plotted points represent technical replicates. ANOVA followed by Tukey’s test. Data are presented as mean *N* = 9 or 10 technical replicates, two independent. **(C)** Effect of desmopressin (dDAVP, 0.1 μM) on the organization of tubular structures by microvascular endothelial HmVEC-L cells using a capillary-morphogenesis assay on a matrix gel coated substrate. Representative images of capillary-like tubular structures after 16 h treatment using vehicle or dDAVP (right, ×100 magnification). Unpaired *t*-test. Box and whiskers (Min to Max). *N* = 10 technical replicates, three independent experiments. **(D)** Proliferative effect of tumor conditioned medium (TCM) from MG-63 cultures on HMEC-1 cells and inhibition of endothelial cell growth after incubation with bevacizumab (BEVA, 25 μg/ml) or its combination with desmopressin (dDAVP, 1 μM). Kruskal–Wallis followed by Dunn’s test. Data are presented as mean ± SEM. *N* = 8–32 technical replicates, four independent experiments. *p < 0.05, **p < 0.01, ***p < 0.001, ****p < 0.0001.

Although we have previously characterized the direct effects of dDAVP on AVPR2-expressing OSA cells (20), its activity on microvascular endothelial cells remains less well-understood, particularly in the context of OSA-driven neovascularization and tumor–vascular crosstalk. Therefore, we investigated the effects of dDAVP on key cellular processes that are critically involved in angiogenesis. From a pharmacological perspective, dDAVP is a highly selective clinically used agonist for AVPR2 with minimal off-target pharmacology. Our study incorporated a pharmacologically relevant concentration of 100 nM (cita), together with an intermediate (1 μM) and an experimental supraphysiological (10 μM) concentration. This range was selected primarily to gain mechanistic insight into dDAVP activity, enabling a more comprehensive characterization of cellular responses and the evaluation of potential concentration-dependent effects. After angiogenic stimulation, endothelial cells from existing vessels enter the cell cycle and proliferate (33). In this experimental context, microvascular cell proliferation was inhibited by dDAVP in a concentration-dependent manner, reducing growth by up to 60% at higher concentrations. It is worth noting that concentrations in the nanomolar range were sufficient to cause a significant mitigation on HMEC-1 growth (Figure 3A). It is known that proliferating endothelial cells then migrate toward the angiogenic gradient. In a 3D chemotaxis assay dDAVP, at 1 and as well 0.1 μM concentrations, significantly inhibited directional migration of microvascular endothelial cells following FBS-gradient (Figure 3B). Finally, endothelial cells undergo morphogenesis by organizing into tubular structures, establishing luminal architecture, and assembling into functional microvascular networks. To evaluate the impact of the repurposed agent on this critical angiogenic process, a matrix gel-based morphogenesis assay was performed using the microvascular endothelial cell line HmVEC-L. As shown in Figure 3C, exposure to 100 nM dDAVP resulted in a significant reduction in the capacity of microvascular endothelial cells to form capillary-like structures. In this context, the responsiveness observed across all endothelial models and angiogenesis-related endpoints at physiologically relevant low-nanomolar concentrations of dDAVP is consistent with functional AVPR2 expression. To begin exploring tumor–vascular crosstalk and the potential impact of combining dDAVP with the anti-VEGF-A agent BEVA, dual therapy was evaluated in HMEC-1 human microvascular endothelial cells cultured under low-serum conditions and exposed to concentrated tumor-conditioned medium (TCM) derived from MG-63 OSA cells. The addition of serum-free TCM doubled HMEC-1 cell growth compared with low-serum control cultures. BEVA alone had no effect on endothelial growth under low-serum conditions. However, treatment with BEVA significantly reduced the growth of TCM-stimulated microvascular cells by 59%, completely abrogating the growth-promoting effect induced by MG-63-conditioned medium. Likewise, dDAVP treatment reduced the growth of TCM-exposed endothelial cells by 30%, consistent with its inhibitory activity previously observed in unstimulated endothelial cultures. Furthermore, the addition of dDAVP to BEVA produced a cooperative antiangiogenic response, resulting in an 80% reduction in microvascular cell growth relative to TCM-treated controls. Although the combination regimen achieved the greatest inhibitory effect, differences compared with either monotherapy did not reach statistical significance (Figure 3D).

**FIGURE 4:**
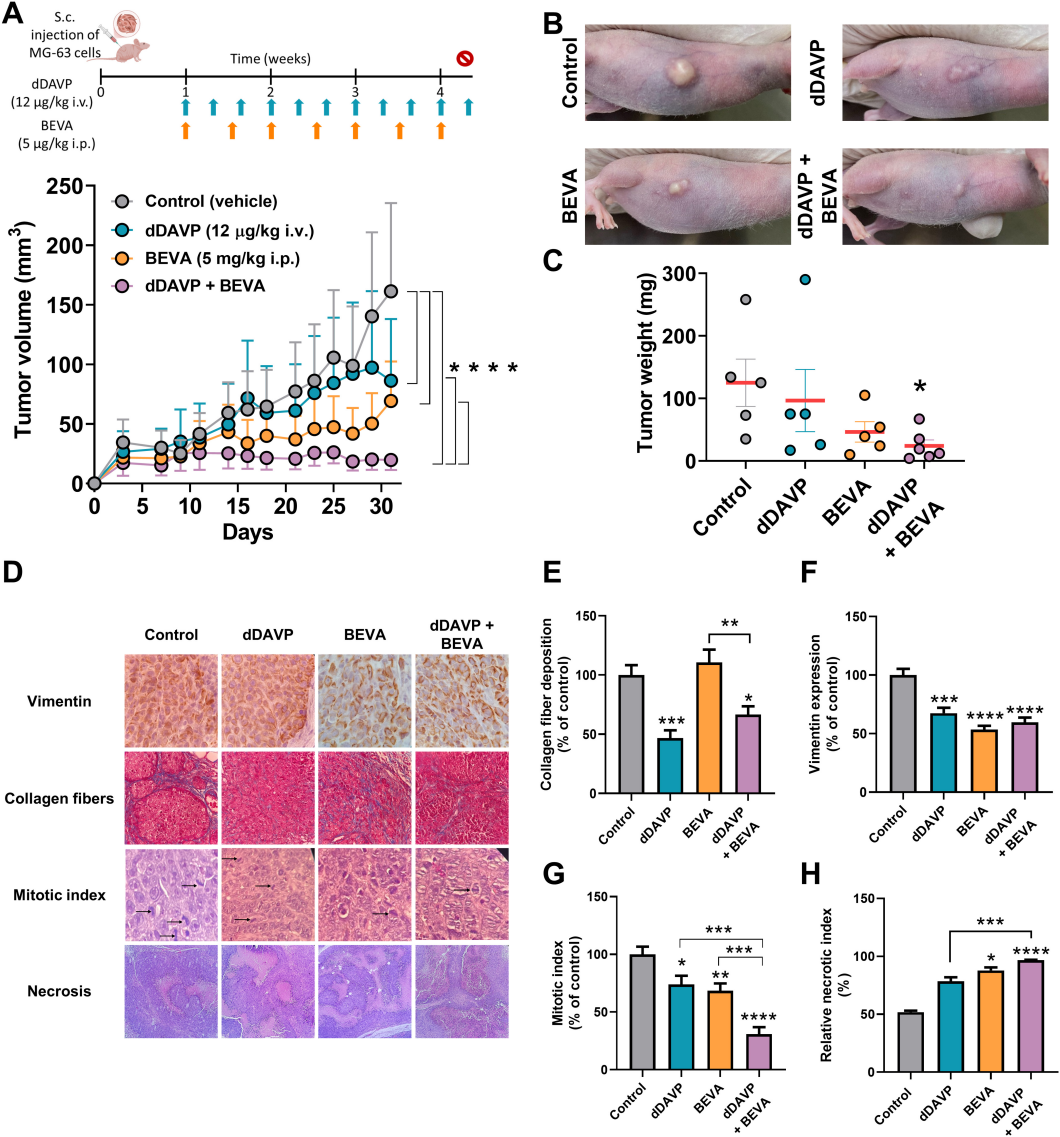
Therapeutic benefits after desmopressin addition to bevacizumab on human osteosarcoma xenograft progression. **(A)** Experimental design used to evaluate *in vivo* effect of desmopressin (dDAVP, 12 μg/kg i.v.) combined with bevacizumab (BEVA, 5 mg/kg i.p.) on tumor progression of MG-63 xenografts growing in athymic mice (Top). Curves represent mean tumor volumes of mice receiving saline vehicle (control), dDAVP, BEVA or dDAVP plus BEVA over time (middle). *N* = 5 or 6 animals per experimental group, one independent experiment. Statistical significance corresponds to tumor growth rates analysis which represents the slopes of the linear regressions of tumor volumes between days 1 and 29. ANOVA followed by Tukey’s test. **(B)** Representative pictures of nude mice bearing osteosarcoma xenografts belonging to different experimental groups at day 31 are shown (bottom). **(C)** Weight of osteosarcoma primary lesions after necropsy. Plotted points represent animals. Kruskal–Wallis followed by Dunn’s test. **(D)** Representative images of vimentin expression assessed by immunohistochemistry (×1,000 magnification), Masson’s trichrome staining of collagen fibers (×400 magnification), mitotic bodies pointed out by black arrows in H&E stained slides (×400 magnification), and necrosis assessment in H&E stained tumor sections (×100 magnification). **(E)** Analysis of collagen fiber deposition. *N* = 5 or 6 high power fields (HPF) per biological replicate. ANOVA followed by Tukey’s test. **(F)** Histologic assessment of vimentin expression. *N* = 10–12 HPF per biological replicate. ANOVA followed by Tukey’s test. **(G)** Quantification of mitotic bodies. *N* = 3 or 4 HPF per biological replicate. ANOVA followed by Tukey’s test. **(H)** Relative necrotic index. *N* = 2 semi-serial complete sections per biological replicate. Kruskal–Wallis followed by Dunn’s test. Data is shown as percentage of control and presented as mean ± SD except for **(C,E)** that is presented as mean ± SEM. *p < 0.05, **p < 0.01, ***p < 0.001, ****p < 0.0001.

Finally, to continue elucidating the dual cytostatic and angiostatic effects of dDAVP on OSA, the potential combination of this repurposed hemostatic drug with the antiangiogenic agent BEVA was evaluated on the progression of aggressive MG-63 xenografts. Once tumors were established and outgrowth was confirmed, treatment schedules using dDAVP (12 μg/kg i.v., three doses per week), BEVA (5 mg/kg i.p., two doses per week), or its combination began. Sustained dDAVP plus BEVA dual therapy blocked tumor growth, slowing the tumor progression rate by nearly 90%, improving the effect of the monotherapies alone (Figure 4A). A significant but partial efficacy was observed for the monotherapies dDAVP and BEVA. Representative tumor-bearing animals and quantification of tumor weight from different experimental groups are depicted in Figures 4B,C, respectively. All treatments were well tolerated, especially the combination. No overt toxicity was observed under the tested conditions (Supplementary Figure 2C). To deepen our understanding of the mechanisms associated with the anti-OSA activity of the combined therapy, we evaluated at the tumor histological level four markers of clinical relevance associated with aggressiveness and therapy response (Figure 4D). First, it is known that collagen deposition supports OSA proliferation and tumorigenesis (34). The presence of this dense collagenous stroma, the so-called desmoplastic response, is deeply associated with poorer immune infiltration and high angiogenic activity (35). Moreover, vimentin is a type III intermediate filament protein and mesenchymalization marker, associated with malignant cell motility and cytoskeletal remodeling (36, 37). Histopathological analysis of tumor samples revealed that combined treatment of dDAVP and BEVA significantly decreased collagen fiber deposition in the tumor stroma and vimentin expression in the cytoplasm of OSA cells, both markers of tumor aggressiveness (Figures 4E, F). It is worth noting that the inhibitory effects observed in the combined group in the collagen fiber deposition analysis were mainly attributed to the individual activity of dDAVP or BEVA, respectively. Taking into account that elevated mitotic counts in OSA are linked to a worse prognosis (38), mitotic index was assessed in resected tumors from different experimental groups. Although all tested therapies achieved significant reductions on mitotic counts, dDAVP addition to BEVA yielded the most potent effect, inhibiting the amount of mitosis by 69.1% in comparison to the control group (Figure 4G). Sustained dDAVP or BEVA as monotherapies were linked to a significant but partial reduction in the proliferating status of OSA cells within the tumor, reducing the number of mitotic figures by 26.1 and 31.5%, respectively. Finally, pathological estimation of tumor necrosis after therapy is essential for patients with OSA (39, 40). Thus, necrosis was assessed for all experimental groups by image analysis. Once again, after necrosis assessment, tumors from animals treated with the combined dDAVP + BEVA regimen exhibited a marked therapeutic response, duplicating the relative necrotic index in comparison to the control group (Figure 4H). Gross necrotic fraction assessment is also depicted in Supplementary Figure 4, confirming a significant impact of dual therapy on tumor necrosis.

## 4 Discussion

To our knowledge, this is the first study to report on the angiostatic and antimetastatic activity of selective AVPR2 agonist and hemostatic compound dDAVP in OSA models, as well as its therapeutic benefits in combination with antiangiogenic agent BEVA. First, exploratory and hypothesis-generating analyses of pan-sarcoma and OSA-specific transcriptomic datasets revealed a potential favorable prognostic association with elevated tumor AVPR2 expression. Moreover, AVPR2 expression in sarcomas was inversely correlated with a broad panel of aggressiveness-related markers linked to key biological processes, including cell survival, cell-cycle progression, evasion of apoptosis, metastatic dissemination, and tumor angiogenesis. We further demonstrated that dDAVP inhibits endothelial chemotaxis, tube formation, and microvascular cell growth *in vitro*, all of which represent critical processes in tumor angiogenesis. *In vivo*, dDAVP also impaired pulmonary metastatic dissemination and altered OSA-driven early vascularization. Finally, the addition of dDAVP to the VEGFA-targeting agent BEVA resulted in reduced tumor progression, providing experimental support and a biological rationale for further investigation of this potential dual antiangiogenic therapeutic strategy in OSA.

The observed antitumoral effects of dDAVP in OSA are in line with previous results reported by our research group and others, on different aggressive types of cancer, including glioma, lung, prostate, breast and colorectal cancers (16, 19, 41–43). Mechanistically, the antineoplastic activity of dDAVP has been associated with a broad spectrum of AVPR2-mediated effects in tumor cells, including induction of mitochondrial dysfunction (41), inhibition of urokinase-type plasminogen activator–dependent invasion (43) chemopotentiation (16) and promotion of the proteolytic conversion of plasminogen into angiostatin (44), a well-characterized endogenous inhibitor of angiogenesis. Particularly in OSA, it was previously reported that dDAVP exerts strong and selective cytostatic activity on AVPR2-expressing malignant cells, lacks significant antitumor effects on AVPR2-negative cells such as the U2OS model, and triggers apoptosis through antiproliferative cAMP-PKA pathways associated with canonical receptor activation (20). Although the AVPR2-dependence of the agents direct activity on OSA cells has been previously addressed, expanding these studies, particularly within further vascular models and events, including AVPR2 knockdown, chemical blockade of AVPR2 via co-incubation with specific receptor antagonists such as tolvaptan, and functional rescue experiments, could strengthen our mechanistic understanding of the therapeutic agent under study.

In addition to its inhibitory effect on OSA growth, our findings suggest a potential angiostatic activity on microvascular cells and an attenuation of OSA-related neovascularization. As previously stated, BEVA has proven partial clinical benefits across different pediatric cancers, including OSA. However, accumulating evidence suggests that targeting angiogenesis at a single step may be insufficient to achieve a sustained antitumor response, and that multi-step inhibition through complementary antiangiogenic strategies may provide improved therapeutic outcomes (45). Repurposed dDAVP may exert a dual antitumor effect through AVPR2 activation in both malignant and endothelial cells. Beyond its direct cytostatic and angiostatic activities, this response may also be mediated indirectly through the release of vascular-derived effectors capable of modulating tumor progression and angiogenesis. Agonist activation of endothelial AVPR2 promotes rapid exocytosis of Weibel–Palade bodies and subsequent release of the hemostatic glycoprotein von Willebrand factor, amongst other molecules. Of importance to this study, although is a multimeric protein with complex biological roles, it has been reported that von Willebrand factor, in complex with factor VIII, can promote apoptosis in OSA cell lines such as MG-63 and Saos-2 (46). Moreover, von Willebrand factor has been implicated in vascular normalization under pathological conditions, as endothelial von Willebrand factor deficiency promotes VEGFR-2–dependent angiogenesis (47). Consistent with this concept, von Willebrand disease is strongly associated with angiodysplasia, a vascular disorder characterized by fragile and aberrant blood vessels, particularly in the gastrointestinal tract, which has been linked to dysregulated angiogenic signaling (48). Moreover, is it known that the von Willebrand factor has a predictive value for response to bevacizumab in aggressive tumors such as recurrent glioma (49). Considering the direct cytostatic and angiostatic effects of dDAVP, combination of this repurposed agent with VEFGA-blockage by BEVA could favor the disruption of protumoral OSA-vascular crosstalk and a cooperative suppression of mitogenic potential of OSA cells. These complementary mechanisms may account for the combinatorial benefits observed in this study, including reduced mitotic counts, impaired xenograft progression, and increased tumor necrosis.

Hemorrhages and other adverse drug reactions associated with BEVA usage in cancer patients are relatively common (11). Among the most frequently reported adverse events are posterior reversible leukoencephalopathy syndrome, gastrointestinal perforation, and cerebrovascular complications, including ischemic stroke and cerebral hemorrhage (12, 50). Addressing the need for dose reduction and safer therapeutic alternatives, particularly in pediatric populations, metronomics has emerged as a promising therapeutic strategy (51). By combining drug repurposing with the sustained administration of low doses of cytotoxic agents, this approach aims to achieve long-term control of tumor progression while minimizing adverse effects and reducing implementation costs, making it particularly attractive for low- and middle-income countries with severe economic constraints (52). In this context, beyond potentially enhancing antitumor efficacy, a metronomic maintenance regimen incorporating dDAVP administration in combination with sustained low-dose BEVA could allow de-escalation of the α-VEGF-A monoclonal antibody dosage, thereby contributing to the reduction of BEVA-related adverse effects.

This study provides promising biomedical evidence proposing, for the first time, a combinatorial rationale for the management of OSA through the use of the repurposed drug dDAVP in addition to the well-established anti-angiogenic agent BEVA. To this end, multiple *in vitro*, *in silico*, and *in vivo* approaches were implemented, employing several well-characterized human and murine models of OSA as well as microvascular endothelial cells that have been widely applied in translational research on OSA therapeutics, testing of antiangiogenic strategies, and tumor vascular biology (21, 22, 26, 53). To characterize the anti-OSA therapeutic activity on primary tumor growth, dissemination, and tumor-associated neovascularization, both immunocompetent and xenogeneic models were used, and clinically relevant dosing schemes were applied, highlighting the translational potential of the findings. In particular for dDAVP, a human clinically-validated dose of 1 μg/kg administered i.v. was used as reference for human-to-animal dose conversion. This dosage has been previously employed in adjuvant oncological settings (54) and, notably, in pediatric populations (55, 56), with a favorable safety and tolerability profile. Moreover, therapeutic responses in tumor tissues were assessed using histopathological biomarkers with recognized clinical relevance in OSA management, including mitotic body count, vimentin expression, tumor necrosis, and desmoplasia (34, 37–39). The latter observation warrants further discussion. In this regard, extracellular matrix (ECM) dysregulation and aberrant collagen deposition are recognized contributors to OSA tumorigenesis, progression, and therapeutic resistance (34). Furthermore, a dense collagen-rich stroma has been associated with increased angiogenic activity and reduced immune cell infiltration, both of which correlate with adverse clinical outcomes (35). In the present study, combined dDAVP plus BEVA therapy reduced stromal collagen deposition within MG-63 xenografts, an effect that appeared to be driven primarily by dDAVP administration. Given that ECM accumulation in OSA is thought to arise predominantly from stromal mesenchymal populations rather than from tumor cells themselves, this finding should not be interpreted as evidence of direct inhibition of collagen synthesis by OSA cells (57). Instead, the reduction in desmoplasia may reflect indirect consequences of antiangiogenic activity on host-derived stromal remodeling, including diminished endothelial–stromal crosstalk, reduced recruitment and activation of matrix-producing mesenchymal cells, and an overall attenuation of tumor-driven microenvironmental signaling. Beyond a potential effect mediated by its direct angiostatic activity, it is also conceivable that the antitumor effects of dDAVP and agonist activation of tumor-associated AVPR2 may reduce the production of profibrotic mediators, such as IL-6, TGF-β, HIF-1α, and other cytokines implicated in stromal activation and matrix remodeling (58). In this context, further mechanistic studies aimed at elucidating the antidesmoplastic effects of dDAVP in OSA will be important for advancing the translational development of the proposed therapeutic strategy.

While the findings presented herein provide encouraging evidence supporting the therapeutic potential of the proposed strategy, several important limitations should be acknowledged when interpreting the results. First, the influence of the OSA tumor stroma should not be overlooked and, despite their experimental complexity and variability, orthotopic bone tumor models should be considered in future studies with the aim of recapitulating the bone microenvironment as closely as possible (59). Secondly, the combination of dDAVP plus BEVA was evaluated *in vivo* using immunodeficient mice bearing human OSA tumors. The reported mechanisms of action of both dDAVP and BEVA lie at the intersection of not only tumor-specific and vascular events, but also immune-related processes, broadly affecting both cellular and non-cellular components of the complex tumor microenvironment (60). In this context, the implementation of syngeneic models should be considered in future studies in order to evaluate the combined approach in fully immunocompetent animals. In light of the aforementioned limitations, validation of this therapeutic approach in orthotopic and/or immunocompetent OSA models will be required before its translational relevance can be firmly established. It is also important to recognize that the tail-vein experimental metastasis model predominantly assesses pulmonary colonization and metastatic outgrowth, while bypassing the earlier stages of metastatic dissemination from a primary bone tumor. Accordingly, a more comprehensive evaluation of the antimetastatic effects of dDAVP in OSA will require the implementation of spontaneous metastasis models that faithfully recapitulate the full metastatic cascade, including local invasion, intravasation, survival in the circulation, vascular arrest and seeding, and subsequent metastatic expansion within the lung microenvironment (32). Furthermore, the Matrigel plug assay employed to assess early angiogenesis provides only preliminary evidence of altered vascularization following dDAVP treatment. Because hemoglobin content serves as an indirect surrogate marker of angiogenic activity, and given the well-established hemostatic and vascular effects of dDAVP, the observed findings do not unequivocally distinguish inhibition of OSA-driven neovascularization from changes in vascular permeability, perfusion, or blood retention within the matrix plug. Therefore, additional vascular characterization, including CD31 immunostaining, quantitative assessment of microvessel density and morphology, and/or the incorporation of vascular perfusion markers, will be required to more comprehensively define the antiangiogenic effects of dDAVP in this experimental setting. Finally, the analysis of the TARGET-OS dataset should be regarded as an exploratory, disease-specific transcriptomic assessment rather than a definitive validation of AVPR2 as a prognostic biomarker in pediatric OSA. While this resource enables the evaluation of AVPR2 expression in relation to available clinical status information, it does not support robust survival analyses, including hazard ratio estimation, censored Kaplan–Meier analysis, disease-free survival assessment, or comprehensive time-to-event modeling. The lack of complete longitudinal follow-up data therefore represents an important limitation. Consequently, validation in prospective cohorts or independently curated OSA datasets with comprehensive survival annotation will be required to determine whether AVPR2 expression possesses independent prognostic value.

A major limitation of incorporating BEVA into OSA treatment regimens is its potential to impair postoperative wound healing, reflecting the shared molecular and cellular mechanisms that govern both pathological angiogenesis and physiological tissue repair. BEVA exposure before or during primary tumor resection was associated with severe bleeding, deep wound infection and dehiscence. These major postoperative complications were reported in pediatric OSA patients, as well as in other prevalent adult malignancies (10, 61, 62). As previously introduced, von Willebrand factor is a multifunctional protein which is also involved in metastatic resistance (63–65), besides hemostasis and bleeding control (24). Experimental studies using *in vivo* models of tumor surgical manipulation and veterinary clinical trials in dogs with advanced carcinomas, showed that perisurgical administration of dDAVP reduces locoregional and distant spread, and extends the survival of treated animals, respectively (66, 67). Leveraging its hemostatic and antitumoral properties, two clinical trials were conducted to evaluate the safety and therapeutic potential of dDAVP in different oncological settings. First, a phase II dose-escalation study assessed dDAVP as a surgical adjuvant during mammary cancer resection (NCT01606072) (54). Subsequently, a phase I/II clinical trial evaluated tolerability, angiogenic parameters, and hemostatic effects in patients with advanced rectal cancer presenting with bleeding (NCT01623206) (68). In both studies, dDAVP exhibited a favorable safety profile and provided clinical benefits in terms of improved bleeding control, a reduction in circulating tumor cells following surgery, and inhibition of tumor vascular perfusion (54, 68). Taking these observations into account, an additional clinical scenario for the implementation of dDAVP could be its perioperative administration (69), delivered immediately before and after aggressive curative surgery for OSA or other highly vascularized sarcomas in patients receiving BEVA therapy. In this context, when used as a surgical adjuvant, the combined hemostatic and antimetastatic properties of dDAVP could potentially mitigate the increased risk of bleeding and wound-healing complications associated with BEVA treatment, while simultaneously protecting patients from tumor cell dissemination during surgical tumor removal.

In OSA, the development of novel antiangiogenic therapies in general, and strategies targeting the VEGF/VEGFR axis in particular, remains both highly promising and inherently challenging (70). Current experimental approaches and agents that have advanced to clinical evaluation encompass a diverse range of therapeutic modalities, including monoclonal antibodies directed against angiogenic growth factors or their receptors (e.g., BEVA and ramucirumab), receptor fusion proteins (e.g., ziv-aflibercept), pepducins and other sphingosine-1-phosphate receptor antagonists (e.g., KRX-725-II), and multi-target tyrosine kinase inhibitors such as sorafenib and regorafenib, among others (71, 72). Despite the high attrition rate of candidate agents and the considerable molecular, biological, and clinical complexity that characterizes OSA, efforts to develop effective antiangiogenic therapies continue to advance. Accumulating evidence suggests that the rational design and clinical evaluation of selective, mechanistically complementary antiangiogenic strategies are essential to improve therapeutic response rates while minimizing treatment-related toxicity.

In conclusion, our findings provide the first experimental evidence supporting a cooperative therapeutic strategy integrating dDAVP with the anti-angiogenic agent BEVA for the management of OSA. By combining complementary cytostatic, angiostatic, and antimetastatic mechanisms, this dual approach may disrupt protumoral OSA-vascular interactions and enhance overall antitumor efficacy. Importantly, the incorporation of a safe and well-tolerated repurposed compound such as dDAVP offers a rapid, affordable strategy with significant translational potential for a malignancy with persistent unmet clinical needs. Further preclinical evaluation aimed at validating this combinatorial approach in clinically relevant settings are therefore warranted.

## Data Availability

The datasets presented in this study can be found in online repositories. RNA sequencing expression data of tumors from TCGA-SARC and TARGET-OS are publicly accessible at the National Center for Biotechnology Information (NCBI) under accession numbers phs000468.v18.p7 (https://www.ncbi.nlm.nih.gov/) and phs000178 (https://www.ncbi.nlm.nih.gov/). The raw data supporting the conclusions of this article will be made available by the authors, without undue reservation.

## Footnotes

1
http://gepia.cancer-pku.cn

2
https://cistrome.shinyapps.io/timer/

3
https://www.datarus.eu/aplicacion/granmo
