# Supplementary material for: Combined treatment using repurposed synthetic peptide desmopressin and bevacizumab as a potential antiangiogenic strategy in osteosarcoma
Source: Front Med (Lausanne). 2026 Jul 1;13:1864843. doi: 10.3389/fmed.2026.1864843 (PMC13394006; doi:10.3389/fmed.2026.1864843)
Supplement: Supplementary file 1 [file Data_Sheet_1.docx]

Supplementary Material: Combined treatment using repurposed synthetic peptide desmopressin and bevacizumab as a potential antiangiogenic strategy in osteosarcoma

# Supplementary Tables

## Supplementary Table 1

| **Biological process involved** | **Gene signature** | **Correlation with AVPR2** | |
| --- | --- | --- | --- |
|  |  | **R** | ***P* value** |
| Cell cycle progression | CDK1 | -0.38 | 1.8e-10 **** |
|  | GNL3 |  |  |
|  | RRS1 |  |  |
|  | PARP1 |  |  |
| Apoptosis induction | STAT5A | 0.3 | 6.7e-07 **** |
|  | OLFM4 |  |  |
|  | PINK1 |  |  |
| Apoptosis evasion | BIRC5 | -0.28 | 4.3e-06 **** |
|  | API5 |  |  |
|  | BCL10 |  |  |
|  | PRC1 |  |  |
|  | TRAP1 |  |  |
| Metastases | HDAC8 | -0.32 | 1.6e-07 **** |
|  | AMACR |  |  |
|  | DLX2 |  |  |
|  | MKI67 |  |  |
| Angiogenesis | VEGFA | -0.26 | 1.5e-05 **** |
|  | MTOR |  |  |
|  | MEK |  |  |

**Supplementary table 1.** **Pooled correlation analysis between AVPR2 expression in sarcoma tumors and gene signatures of different biological processes relevant in disease progression using GEPIA2.** RNA-sequencing information from The Cancer Genome Atlas (TCGA), sarcoma (SARC) database (N=257). Rho>0 positive correlation, Rho<0 negative correlation. Spearman's rank correlation coefficient. ****p<0.0001.

| **Description** | **Gene markers** | **None** |  |  |  | **Purity** |  |  |
| --- | --- | --- | --- | --- | --- | --- | --- | --- |
|  |  | **Rho** | ***P* value** | **Adjusted *P* value (BH-FDR)** |  | **Rho** | ***P* value** | **Adjusted *P* value (BH-FDR)** |
| Natural killer cell | KIR3DL2 | 0.182 | 3.23e-03 ** | 5.11e-03 ** |  | 0.155 | 1.56e-02 * | 2.69e-02 * |
|  | KIR3DL1 | 0.222 | 3.09e-04 *** | 6.52e-04 ** |  | 0.194 | 2.33e-03 ** | 4.92e-03 ** |
|  | KIR2DL3 | 0.137 | 2.71e-02 * | 3.32e-02 * |  | 0.097 | 1.31e-01 | 1.64e-01 |
|  | KIR2DL1 | 0.125 | 4.32e-02 * | 4.97e-02 * |  | 0.089 | 1.67e-01 | 1.98e-01 |
| M1 Macrophage | IRF5 | 0.243 | 7.79e-05 *** | 1.97e-04 *** |  | 0.178 | 5.29e-03 ** | 1.01e-02 * |
| M2 Macrophage | CD163 | 0.169 | 6.33e-03 ** | 9.62e-03 ** |  | 0.087 | 1.76e-01 | 2.03e-01 |
|  | MS4A4A | 0.25 | 4.73e-05 *** | 1.28e-04 *** |  | 0.199 | 1.77e-03 ** | 3.96e-03 ** |
|  | VSIG4 | 0.146 | 1.87e-02 * | 2.37e-02 * |  | 0.069 | 2.85e-01 | 3.19e-01 |
| TAM | CCL2 | 0.15 | 1.53e-02 * | 2.04e-02 * |  | 0.096 | 1.34e-01 | 1.64e-01 |
|  | IL10 | 0.133 | 3.26e-02 * | 3.87e-02 * |  | 0.048 | 4.53e-01 | 4.78e-01 |
| T cell | CD3E | 0.279 | 5.11e-06 *** | 3.24e-05 **** |  | 0.240 | 1.5e-04 *** | 6.33e-04 *** |
|  | CD2 | 0.258 | 2.56e-05 *** | 8.33e-05 **** |  | 0.218 | 6.11e-04 *** | 1.55e-03 ** |
|  | CD3D | 0.267 | 1.32e-05 *** | 7.17e-05 **** |  | 0.224 | 4.27e-04 *** | 1.16e-03 ** |
| CD8+ T cell | CD8B | 0.262 | 1.88e-05 *** | 7.94e-05 **** |  | 0.225 | 3.91e-04 *** | 1.14e-03 ** |
|  | CD8A | 0.195 | 1.62e-03 ** | 2.80e-03 ** |  | 0.148 | 2.08e-02 * | 3.44e-02 * |
| CD4 + T cell | CD4 | 0.182 | 1.68e-04 *** | 3.99e-04 *** |  | 0.106 | 9.82e-02 * | 1.38e-01 |
| Th1 | TBX21 | 0.308 | 4.09e-07 *** | 5.18e-06 **** |  | 0.279 | 9.55e-06 *** | 6.24e-05 **** |
|  | STAT4 | 0.225 | 2.48e-04 *** | 5.54e-04 *** |  | 0.174 | 6.46e-03 ** | 1.17e-02 * |
| Th2 | STAT5A | 0.304 | 5.85e-07 *** | 5.56e-06 **** |  | 0.258 | 4.53e-05 *** | 2.46e-04 *** |
|  | STAT6 | 0.199 | 1.28e-03 ** | 2.32e-03 ** |  | 0.233 | 2.45e-04 *** | 8.46e-04 *** |
| Th17 | IL17A | 0.011 | 8.56e-01 | 8.56e-01 |  | -0.016 | 8.01e-01 | 8.01e-01 |
| Treg | FOXP3 | 0.093 | 1.35e-01 | 1.43e-01 |  | 0.04 | 5.34e-01 | 5.48e-01 |
|  | STAT5B | 0.155 | 1.24e-02 * | 1.75e-02 * |  | 0.234 | 2.26e-04 *** | 8.46e-04 *** |
|  | CCR8 | 0.163 | 8.34e-03 ** | 1.22e-02 * |  | 0.126 | 5.02e-02 * | 7.63e-02 |
| TAFs | Alpha SMA | 0.150 | 1.56e-02 * | 2.04e-02 * |  | 0.241 | 1.45e-04 *** | 6.33e-04 *** |
|  | Col11A1 | -0.115 | 6.29e-02 * | 7.03e-02 |  | -0.102 | 1.12e-01 | 1.52e-01 |
|  | CD140B | 0.11 | 7.79e-02 * | 8.46e-02 |  | 0.098 | 1.29e-01 | 1.64e-01 |
| Mast cell | CD203C | 0.250 | 4.49e-05 *** | 1.28e-04 *** |  | 0.280 | 8.88e-06 *** | 6.24e-05 **** |
|  | CD35 | 0.210 | 6.48e-04 *** | 1.23e-03 ** |  | 0.146 | 2.22e-02 * | 3.52e-02 * |
|  | CD117 | 0.257 | 2.63e-05 *** | 8.33e-05 **** |  | 0.279 | 9.85e-06 *** | 6.24e-05 **** |
|  | CD45 | 0.186 | 2.66e-03 ** | 4.39e-03 ** |  | 0.12 | 6.07e-02 * | 8.87e-02 |
| CMP | CD34 | 0.414 | 3.46e-12 *** | 6.57e-11 **** |  | 0.415 | 1.39e-11 *** | 2.64e-10 **** |
|  | FC gamma R | 0.029 | 6.4e-01 | 6.57e-01 |  | -0.055 | 3.93e-01 | 4.27e-01 |
|  | SLAMF1 | 0.257 | 2.57e-05 *** | 8.33e-05 **** |  | 0.206 | 1.18e-03 ** | 2.80e-03 ** |
| HSC | EPCR | 0.215 | 4.77e-04 *** | 9.54e-04 *** |  | 0.192 | 2.62e-03 ** | 5.24e-03 ** |
|  | CD49F | 0.292 | 1.64e-06 *** | 1.25e-05 **** |  | 0.297 | 2.33e-06 *** | 2.95e-05 **** |
|  | CD31 | 0.475 | 4.53e-16 *** | 1.72e-14 **** |  | 0.479 | 2.25e-15 *** | 8.55e-14 **** |
|  | CD43 | 0.262 | 1.86e-05 *** | 7.94e-05 **** |  | 0.229 | 3.1e-04 *** | 9.82e-04 *** |

**Supplementary table 2. Correlation analysis between AVPR2 expression in sarcoma tumors and gene markers of different immune and other infiltrating stromal cells using TIMER 2.0.** RNA-sequencing information from The Cancer Genome Atlas (TCGA), sarcoma (SARC) database (N=257). None, correlation without adjustment. Purity, correlation adjusted by purity. Rho>0 positive correlation, Rho<0 negative correlation. Spearman's rank correlation coefficient. Benjamini–Hochberg false discovery rate (BH-FDR) adjusted p-values for both the none-corrected and purity-corrected correlation analyses. *p<0.05, **p<0.01, ***p<0.001, ****p < 0.0001.

| **Description** | **Gene signature** | **Correlation with AVPR2** | |
| --- | --- | --- | --- |
|  |  | **R** | ***P* value** |
| Natural killer cell | KIR3DL2 | 0.22 | 3.7 e-04 *** |
|  | KIR3DL1 |  |  |
|  | KIR2DL3 |  |  |
|  | KIR2DL1 |  |  |
| Macrophage | CD163 (M2) | 0.2 | 1.1e-03 ** |
|  | MS4A4A (M2) |  |  |
|  | VSIG4 (M2) |  |  |
|  | CCL2 (TAM) |  |  |
|  | IL10 (TAM) |  |  |
|  | IRF5 (M1) |  |  |
| T cell | CD3E | 0.26 | 2.2e-05 **** |
|  | CD2 |  |  |
|  | CD3D |  |  |
| CD8+ T cell | CD8B | 0.23 | 2.2e-04 *** |
|  | CD8A |  |  |
| T helper | TBX21 (Th1) | 0.32 | 9.2e-08 **** |
|  | STAT4 (Th1) |  |  |
|  | STAT5A (Th2) |  |  |
|  | STAT6 (Th2) |  |  |
|  | IL17A (Th17) |  |  |
| Treg | FOXP3 | 0.2 | 9.4e-04 *** |
|  | STAT5B |  |  |
|  | CCR8 |  |  |
| TAFs | Alpha SMA | 0.037 | 0.55 |
|  | Col11A1 |  |  |
|  | CD140B |  |  |
| Mast cell | CD203C | 0.25 | 3.9e-05 **** |
|  | CD35 |  |  |
|  | CD117 |  |  |
|  | CD45 |  |  |
| CMP | CD34 | 0.29 | 1.3e-06 **** |
|  | FC gamma R |  |  |
|  | SLAMF1 |  |  |
| HSC | EPCR | 0.41 | 4.7e-12 **** |
|  | CD49F |  |  |
|  | CD31 |  |  |
|  | CD43 |  |  |

**Supplementary table 3. Pooled correlation analysis between AVPR2 expression in sarcoma tumors and gene signatures of different immune and other infiltrating stromal cells using GEPIA2.** RNA-sequencing information from The Cancer Genome Atlas (TCGA), sarcoma (SARC) database (N=257). Rho>0 positive correlation, Rho<0 negative correlation. Spearman's rank correlation coefficient. **p<0.01, ***p<0.001, ****p < 0.0001.

# Supplementary Figures

## Supplementary Figure 1


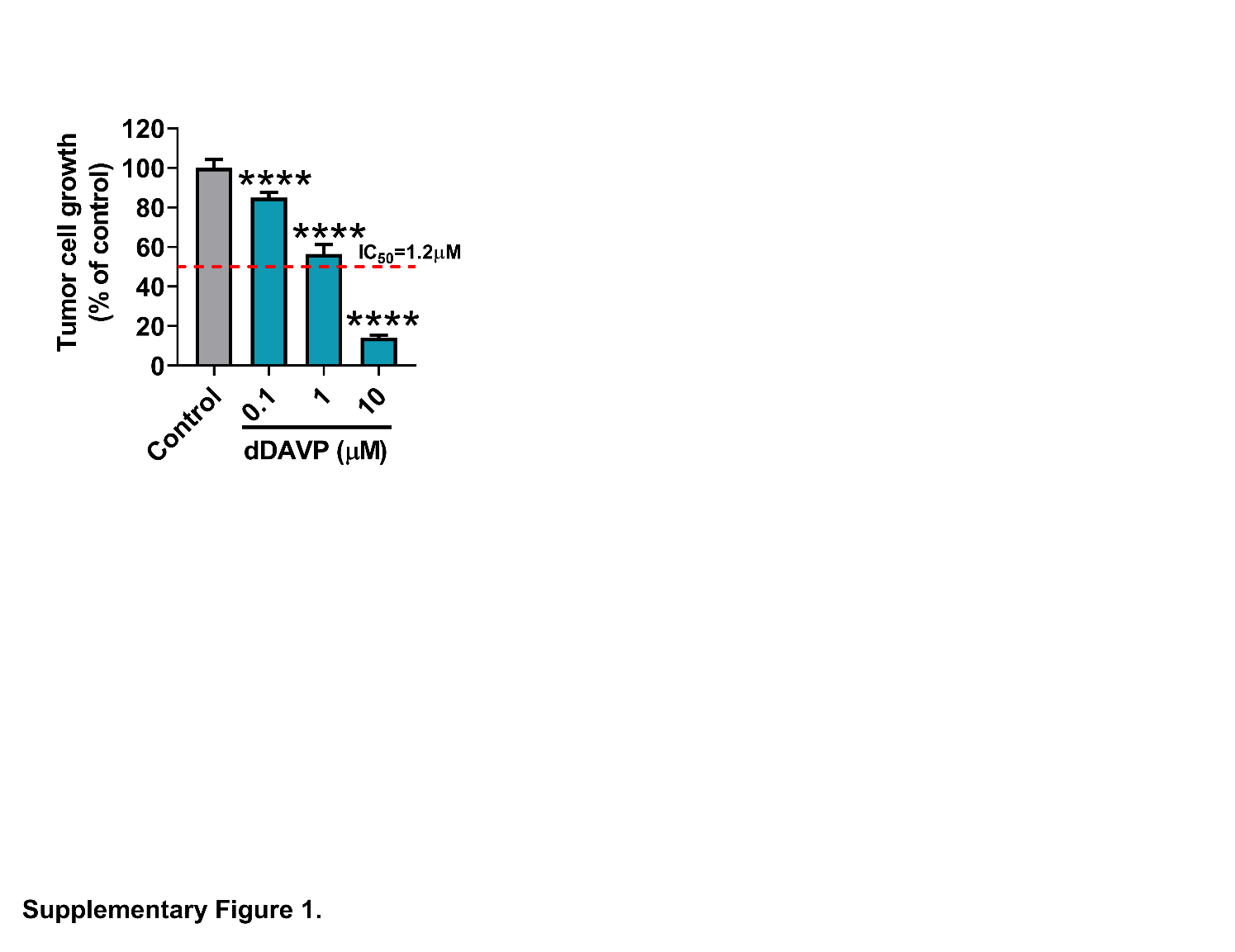


**Supplementary Figure 1.** **Direct cytostatic activity of desmopressin on murine osteosarcoma cells.** Inhibition of cell growth by desmopressin (dDAVP, 0.1–10 μM) on K7M3 cell cultures and calculation of IC50 value (non-linear regression). ANOVA followed by Tukey’s test. Data are presented as mean ± SD. N=16 technical replicates per experimental group, two independent experiments. ****p < 0.0001.

## Supplementary Figure 2


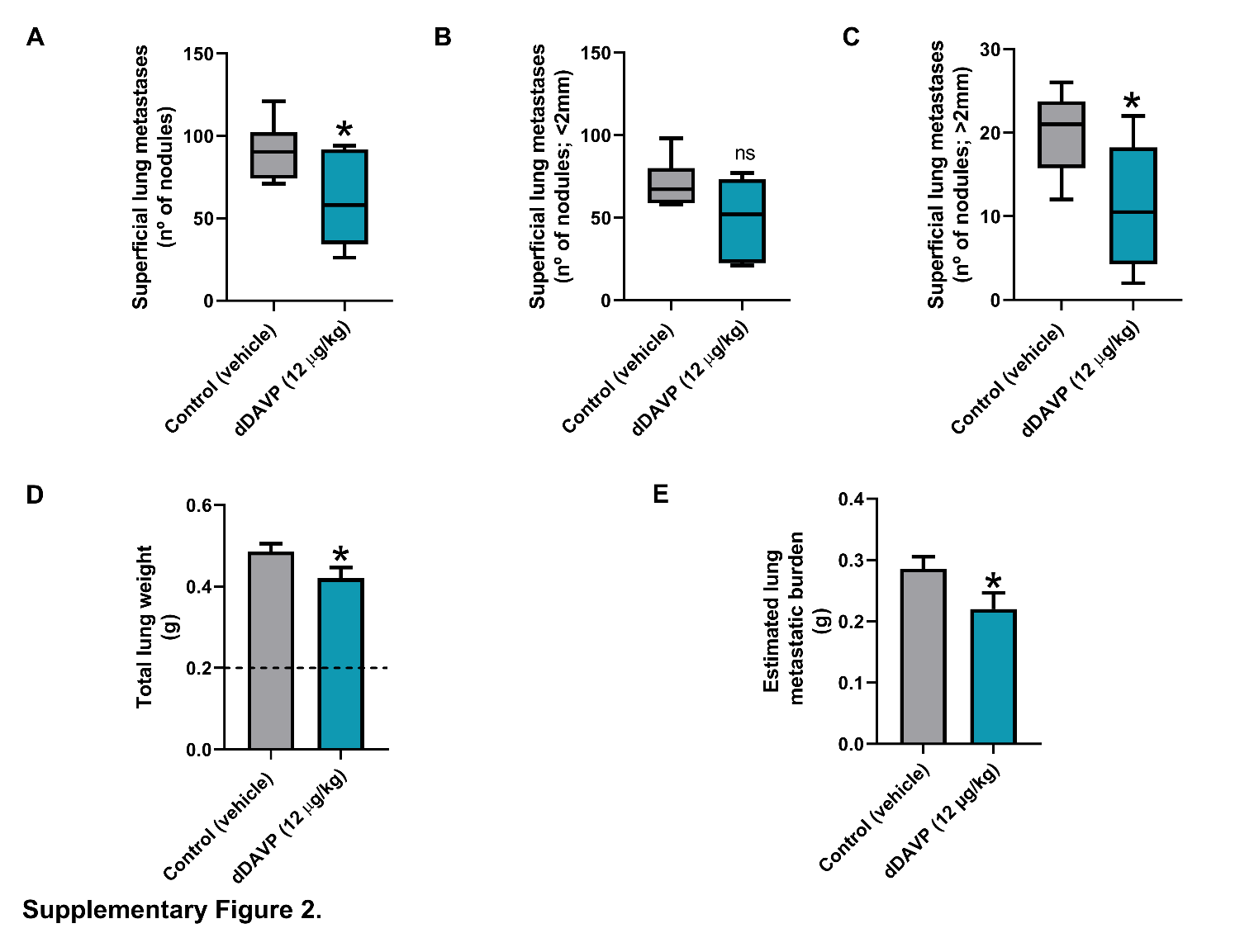


**Supplementary figure 2. *In vivo* effect of desmopressin on pulmonary metastases of K7M3 OSA cells in immunocompetent mice.** N=6 animals per experimental group, one independent experiment. **A)** Quantification of total metastatic nodules. Unpaired t test. Box and whiskers (Min to Max). **B)** Quantification of nodules smaller than 2 milimeters (mm). Unpaired t test. Box and whiskers (Min to Max). **C)** Quantification of nodules bigger than 2 milimeters (mm). Unpaired t test. Box and whiskers (Min to Max). **D)** Lung weight. The dotted line represents the average pulmonary weight of healthy BALB/c mice. Unpaired t test. **E)** Estimated lung metastatic burden. Unpaired t test. *p<0.05.

## Supplementary Figure 3


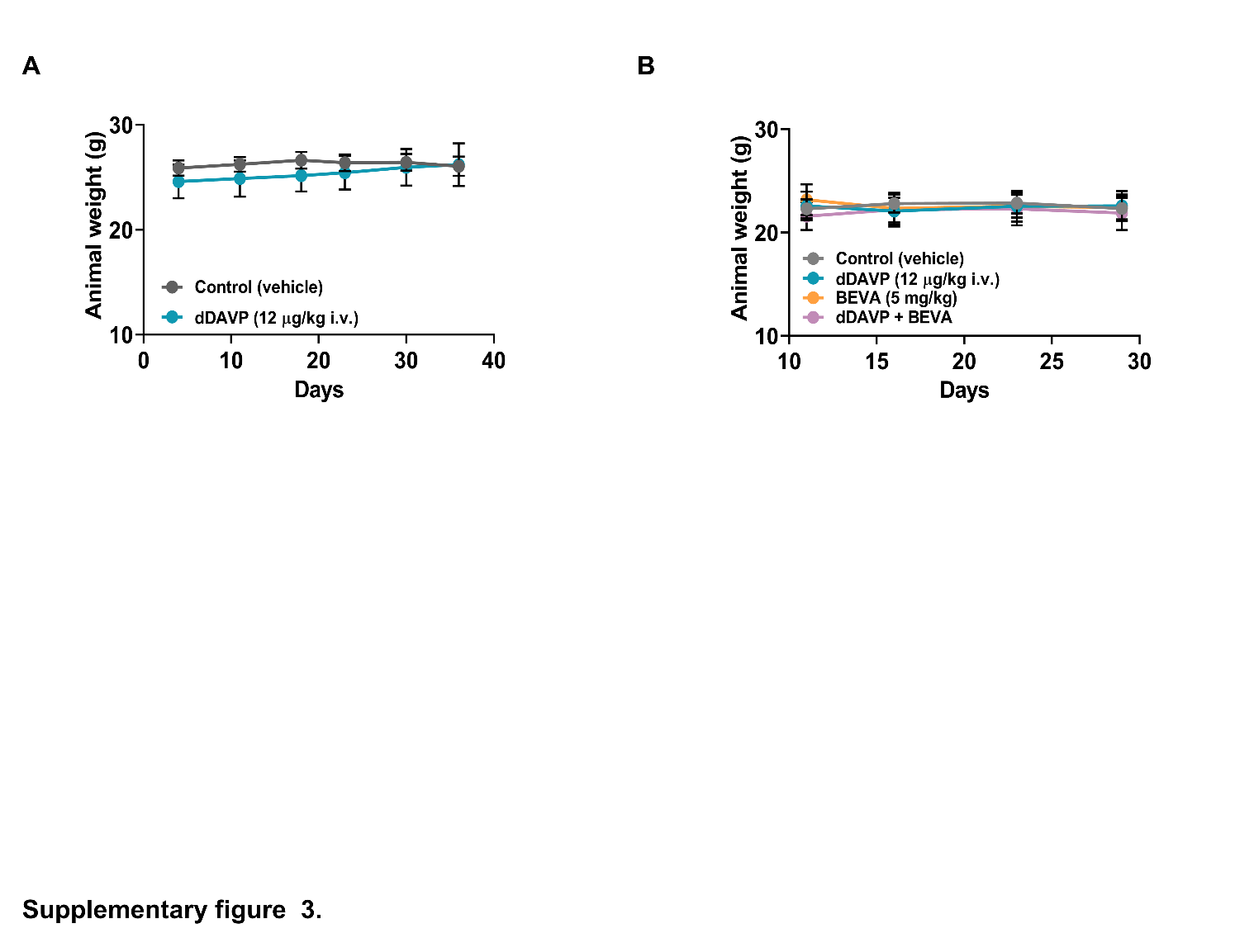


**Supplementary figure 3. Monitoring of animal body weight of long-term *in vivo* protocols. A)** Body weight of animals injected intravenously with K7M3 osteosarcoma cells for the evaluation of the antimetastatic activity of desmopressin (dDAVP). N=6 animals per experimental group, one independent experiment. **B)** Body weight of animals bearing human osteosarcoma MG-63 xenografts during the study of desmopressin (dDAVP) combined with bevacizumab (BEVA) on primary tumor growth. N=5 or 6 animals per experimental group, one independent experiment. For clarity and better visualization of the data, the values of all the Y axes and on the X axes of B) start from 10 g, and 10 days. Data are presented as mean ± SD.

## Supplementary Figure 4


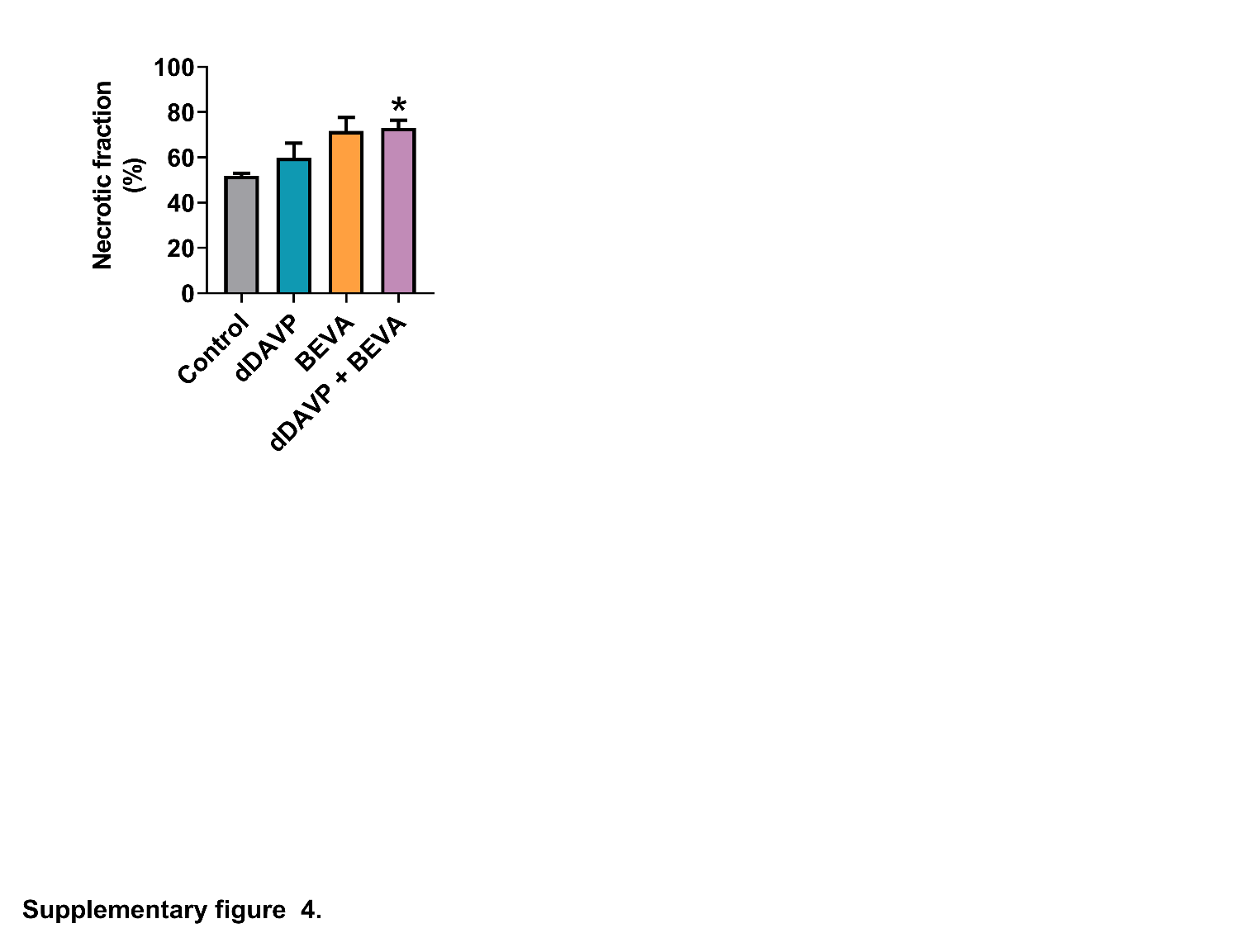


**Supplementary figure 4. Tumor necrosis assessment after desmopressin addition to bevacizumab on human osteosarcoma xenograft progression.** Necrotic fraction corresponds to tumor necrotic rate (TNR) values (for further detail see Materials and Methods section). N=5 or 6 animals per experimental group, one independent experiment. Kruskal Wallis followed by Dunn’s test. Data presented as mean ± SEM.
